# Differences in one-year health outcomes and resource utilization by definition of prolonged mechanical ventilation: a prospective cohort study

**DOI:** 10.1186/cc5667

**Published:** 2007-01-23

**Authors:** Christopher E Cox, Shannon S Carson, Jennifer H Lindquist, Maren K Olsen, Joseph A Govert, Lakshmipathi Chelluri, the Quality of Life After Mechanical Ventilation in the Aged (QOL-MV) Investigators

**Affiliations:** 1 Department of Medicine, Division of Pulmonary and Critical Care Medicine, Duke University, Box 3683, Durham, North Carolina, 27710 USA; 2 Department of Medicine, Division of Pulmonary and Critical Care Medicine, University of North Carolina, 4134 Bioinformatics Bldg, CB# 7020, Chapel Hill, North Carolina, 27599 USA; 3 Center for Health Services Research in Primary Care, VA Medical Center, 11033 Hock Bldg 2424 Erwin Road, Durham, North Carolina, 27705 USA; 4 Department of Biostatistics and Bioinformatics, Duke University, 7020 N. Pavilion Building, Durham, North Carolina, 27710 USA; 5 Department of Critical Care Medicine, University of Pittsburgh School of Medicine 637 Scaife, Pittsburgh, Philadelphia, 15261 USA

## Abstract

**Introduction:**

The outcomes of patients ventilated for longer than average are unclear, in part because of the lack of an accepted definition of prolonged mechanical ventilation (PMV). To better understand the implications of PMV provision, we compared one-year health outcomes between two common definitions of PMV as well as between PMV patients and those ventilated for shorter periods of time.

**Methods:**

We conducted a secondary analysis of prospectively collected data from medical and surgical intensive care units at an academic tertiary care medical center. The study included 817 critically ill patients ventilated for ≥ 48 hours, 267 (33%) of whom received PMV based on receipt of a tracheostomy and ventilation for ≥ 96 hours. A total of 114 (14%) patients met the alternate definition of PMV by being ventilated for ≥ 21 days. Survival, functional status, and costs were measured at baseline and at 2, 6, and 12 months after discharge. Of one-year survivors, 71 (17%) were lost to follow up.

**Results:**

PMV patients ventilated for ≥ 21 days had greater costs ($140,409 versus $143,389) and higher one-year mortality (58% versus 48%) than did PMV patients with tracheostomies who were ventilated for ≥ 96 hours. The majority of PMV deaths (58%) occurred after hospital discharge whereas 67% of PMV patients aged 65 years or older had died by one year. At one year PMV patients on average had limitations in two basic and five instrumental elements of functional status that exceeded both their pre-admission status and the one-year disability of those ventilated for < 96 hours. Costs per one-year survivor were $423,596, $266,105, and $165,075 for patients ventilated ≥ 21 days, ≥ 96 hours with a tracheostomy, and < 96 hours, respectively.

**Conclusion:**

Contrasting definitions of PMV capture significantly different patient populations, with ≥ 21 days of ventilation specifying the most resource-intensive recipients of critical care. PMV patients, particularly the elderly, suffer from a significant burden of costly, chronic critical illness and are at high risk for death throughout the first year after intensive care.

## Introduction

Intensive care is expensive, particularly for those who require mechanical ventilation [1]. Because respiratory failure incidence increases markedly after age 60 years, the aging of the US population will probably strain the health care system's capacity to meet future critical care demands [2,3]. Patients who require prolonged mechanical ventilation (PMV) are a growing group of patients who provoke particular controversy with regard to their uncertain long-term outcomes and disability as well as their disproportionate resource utilization [4].

Clinical decision making and policy making regarding PMV provision is challenging because of the medical literature's confusing array of PMV definitions, ranging from as few as 24 hours to more than 29 days [5,6]. As a result, some have reported that PMV patients experience poor survival, low quality of life, diminished functional status and poor cognitive functioning, and require substantial postdischarge care giving, whereas other have demonstrated a survival benefit from PMV [4,7-10]. A consensus group recently recommended defining PMV as a total duration of ventilation of 21 days or more [11]. Many investigators favor Medicare's definition of tracheostomy and ventilation for at least four days (diagnosis related groups [DRGs] 541 and 542; formerly DRG 483) because diagnostic codes facilitate data extraction from secondary databases and permit linkage to payment data. However, the earlier timing of tracheostomy placement may be altering the composition of the DRG 541/542 population [12-14]. Defining PMV by ventilator days, therefore, may be more specific for the most resource-intensive critically ill patients, in addition to having more meaning for the practicing clinician [4].

There also are problems with the PMV literature that extend beyond definition. Namely, most data on the long-term health experiences of PMV patients are cross-sectional and do not include comparisons with those who are ventilated for shorter periods of time [15]. Additionally, no prospective studies of PMV patients, to our knowledge, have attempted to address the methodological shortcomings associated with this population's high rates of postdischarge death and dropout in longitudinal analyses of health outcomes [16].

Together, these limitations represent a notable barrier to understanding how different clinical factors affect outcomes and the rate of recovery, assessing the overall cost-effectiveness of PMV, meeting the informational needs of patients and families, and informing decisions regarding interventions in this expanding patient group [12,17,18]. To address these issues, we performed novel analyses of previously collected data from a prospective cohort of critically ill patients, with the following *a priori *hypothesizes: identification of PMV patients using DRG 541/542 is less specific for selecting a resource-intensive patient group than a definition of ≥ 21 days of mechanical ventilation; and patients with PMV have higher mortality rates, worse quality of life, and greater functional limitations at one year than patients requiring shorter periods of mechanical ventilation.

## Materials and methods

### Patients, study site, and procedures

These analyses are based on data that were originally collected at the University of Pittsburgh Medical Center in the QOL-MV (Quality of Life After Mechanical Ventilation in the Aged) study, a one-year prospective cohort study whose protocol has been described elsewhere [19,20]. Briefly, all patients aged 18 years or older who received mechanical ventilation for ≥ 48 hours in the medical, general surgical, trauma, and neurologic intensive care units (ICUs) were screened for enrollment. Exclusion criteria were lack of English fluency, receipt of a solid organ transplant, prisoners, baseline chronic ventilation, and hospital transfers ventilated for more than 24 hours before arrival. Data were collected between 1997 and 2000.

### Data collection

**Figure 1:**
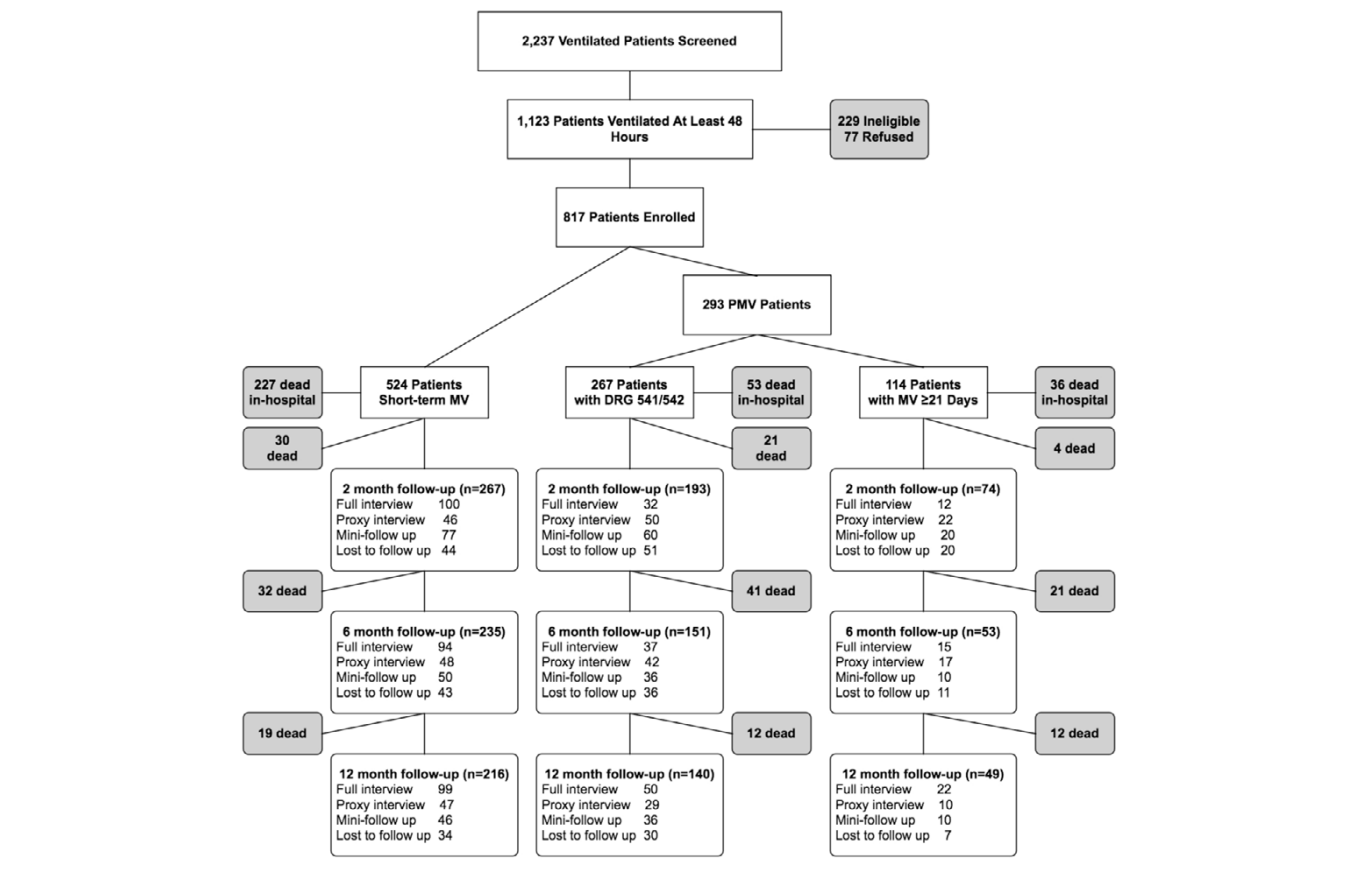
Flowchart of participants in the study by DRG 541/542 status. Diagram demonstrates enrollment of 817 patients into this prospective study. DRG, diagnosis related group.

In baseline in-hospital interviews, study staff recorded patients' sociodemographics, prehospital functional status and physical function aspects of quality of life, medical comorbidities, length of ICU and hospital stay, day one Acute Physiology and Chronic Health Evaluation III score, diagnostic category (medical, surgical, trauma, or other), and admitting source (emergency room, ward transfer, postoperative, outside transfer, other; Figure 1) [21-25]. In postdischarge follow-up interviews (at 2, 6, and 12 months) patient vital status, quality of life, functional status, and need for care giver assistance were recorded. Approximately one-third of interviews involved the use of proxy responses by patients' designated informal care givers because of patients' severe illnesses or degree of cognitive dysfunction. Mini follow-ups (at 2, 6, and 12 months) were abbreviated interviews conducted in those patients or care giver proxies who were unable or unwilling to complete the full follow-up protocol.

Quality of life was measured using the Short Form 36-Item questionnaire (SF-36), a questionnaire for which there is evidence of validity among ICU survivors [26]. We reported values for the SF-36's physical function and role physical domains preferentially because of their objective nature and amenability to proxy assessment. Functional status was measured as the number of dependencies in activities of daily living (ADLs) and instrumental activities of daily living (IADLs) [22,24]. We quantified medical comorbidities using the Charlson index, a validated measure with higher scores indicating greater burden of illness [21]. Mortality was recorded from medical records, physician reports, death certificates, and the Social Security Death Index [27]. Costs were obtained by multiplying hospital charges by Medicare cost to charge ratios and adjusted to 2005 US$ using the medical component of the consumer price index [28].

### Outcomes

Our primary outcomes were one-year survival, functional status, quality of life, and hospital costs. The main group of interest was patients with PMV, which we defined in two different ways: DRG 541/542 (mechanical ventilation for ≥ 96 hours with placement of tracheostomy for non-head and neck diagnoses either with [DRG 541] or without [DRG 542] an operative diagnosis) and ventilation for ≥ 21 days total (with ventilation discontinued for no more than 48 hours). We defined a comparative short-term mechanical ventilation group as those ventilated for ≥ 48 hours who did not meet either PMV definition.

Regarding DRGs, Medicare reimburses US acute hospital care based on adjustment of a base payment by one of these 526 condition-specific weights. This condition-adjusted DRG payment can be further adjusted for hospital-specific factors such as local wage, participation in medical education, and volume of indigent care provided. DRG 541/542 has a very high relative weight, meaning that reimbursement is higher than for many other common conditions.

### Statistical analyses

We addressed the problem of missing data due to death and disability common to longitudinal critical care outcomes studies by using multiple imputation and linear mixed-effects models. In contrast to single imputation methods (for example, last observation carried forward or mean substitution), multiple imputation replaces each missing value by multiple values [29]. We chose not to use a single imputation method because it would not have accurately reflected the uncertainty that is imposed by filling in a single missing value, leading to standard errors that are too small. Instead, multiple imputation reflects missing data uncertainty and results in multiple versions of a complete dataset. Each of these multiple versions are analyzed using the same model, and the estimates and standard errors from each model are combined using Rubin's rules [30]. The combined estimates incorporate both within- and between-imputation variability, and therefore they reflect missing data uncertainty. In addition, linear mixed-effects models are particularly useful for longitudinal data because each patient can have an unequal number of observations, although individuals with more observations will contribute more precise information to parameter estimation [31]. Both of these methods assume that the reason for dropout is 'ignorable' [30].

We first compared baseline characteristics between patient groups (DRG 541/542 versus short-term ventilation) using χ^2 ^tests for dichotomous variables and two-sample *t*-tests for continuous variables. For longitudinal analyses involving hospital survivors, ten multiply imputated datasets were generated under a multivariate normal model using Markov chain Monte Carlo methods in the SAS function PROC_MI. We then fitted linear mixed-effects models using the SAS function PROC_MIXED [16]. Our linear mixed models incorporated potentially confounding baseline variables found to have an association (*P *< 0.20) with both DRG 541/542 status and the outcome of interest, including preadmission Charlson score, preadmission IADLs, admission diagnosis, admission source, education level, age, and APS. These adjusted models allowed us to compare PMV group-level growth curves of quality of life and functional status scores over the course of one year and to determine the extent to which these trajectories were modified by patient characteristics. The mixed-effects models were fitted to the ten imputed datasets, and parameter estimates and standard errors were combined using the SAS function PROC_MIANALYZE.

We also contrasted one-year survival between groups by PMV status (DRG 541/542 versus short-term ventilation) using a piecewise-constant time-varying nonproportional hazard model to generate hazard ratios and 95% confidence intervals for PMV status, a variable that we found to violate the proportional hazards assumption when tested using scaled Schoenfeld residuals and log-log plots [32]. We included in the model preadmission IADLs and Charlson score, day one APS, admitting service, age, and education status, because these variables exhibited group-level differences of statistical (*P *< 0.20) or clinical significance.

Stata 9 (Statcorp, College Station, TX, USA) and SAS 9.1 (SAS Institute Inc., Cary, NC, USA) were used in analyses. The institutional review board of the University of Pittsburgh approved the original protocol, and Duke University's institutional review board approved this secondary analysis.

## Results

### Baseline sociodemographics and clinical characteristics

A total of 817 patients drawn from a potential pool of 1123 patients ventilated for 48 hours were included in the study, of whom 267 (33%) met our study criteria for DRG 541/542 (Figure 1). A total of 114 (14%) of the 817 patients were ventilated for ≥ 21 days, 88 (77%) of whom received tracheostomies and therefore also met the definition of DRG 541/542. The median age was around 65 years in both groups and most patients were male, white, lived at home before admission, and were treated in a medical ICU (Table 1). Compared with patients ventilated short term, DRG 541/542 patients had less medical comorbidities, fewer dependencies in ADLs and IADLs, and better preadmission SF-36 physical function scores (all *P *< 0.02). Sociodemographics, work status before admission, and admission source were not significantly different between persons ventilated short term and those ventilated for prolonged periods (*P *> 0.05).

**Table 1:** Baseline sociodemographics and clinical characteristics

Characteristic	Short-term MV (*n *= 524)	DRG 541/542 (*n *= 267)	MV ≥ 21 days (*n *= 114)
Age	65 (49 to 75)	66 (45 to 75)	66 (47 to 74)
Age group (years)			
≤ 34	57 (11%)	42 (16%)	12 (11%)
35–54	124 (24%)	59 (22%)	33 (29%)
55–64	79 (15%)	26 (10%)	10 (9%)
65–74	121 (23%)	68 (25%)	32 (28%)
75–84	110 (21%)	64 (24%)	25 (22%)
≥ 85	33 (6%)	8 (3%)	2 (2%)
Female	255 (48%)	110 (41%)*	45 (39%)
Race^a^			
Black	87 (16%)	35 (13%)	19 (17%)
White	435 (83%)	231 (87%)	94 (82%)
Other	2 (1%)	1 (1%)	1 (1%)
Marital status			
Married	257 (49%)	133 (51%)	66 (59%)
Unmarried	259 (51%)	126 (49%)	45 (41%)
Education			
High school or less	256 (86%)	159 (73%)*	69 (72%)
More than high school	140 (14%)	59 (27%)	27 (28%)
Income			
< $20,000	139 (48%)	86 (57%)	33 (48%)
≥ $20,000	149 (52%)	64 (43%)	36 (52%)
Residence before hospitalization			
Home	455 (87%)	251 (94%)*	106 (93%)
Rehab facility	10 (2%)	3 (1%)	0 (0%)
Nursing facility	55 (10%)	11 (4%)	7 (6%)
Other	4 (1%)	2 (1%)	1 (1%)
Work status before hospitalization			
Employed	103 (21%)	63 (24%)	26 (24%)
Student	10 (2%)	5 (2%)	1 (1%)
Homemaker	50 (9%)	24 (9%)	10 (9%)
Retired	224 (46%)	108 (40%)	44 (40%)
Unemployed	68 (14%)	43 (16%)	17 (16%)
Disabled	36 (7%)	8 (3%)	11 (10%)
Charlson Index	2.4 (2.6)	1.8 (2.3)*	2.2 (2.7)
Missing	1 (1%)	0 (0%)	0 (0%)
ADLs	1.4 (2.1)	0.8 (1.7)*	1.0 (1.7)^†^
Missing	84 (17%)	41 (12%)	16 (14%)
IADLs	2.9 (2.9)	2.0 (2.8)*	2.2 (2.8)^†^
Missing	146 (28%)	56 (21%)	26 (23%)
SF-36 physical function	48 (39)	62 (38)*	56 (40)^†^
Missing	135 (26%)	56 (21%)	22 (19%)
Primary admission diagnosis			
Medical	350 (67%)	142 (53%)*	70 (61%)
Respiratory	140 (40%)	54 (38%)	29 (42%)
Cardiovascular	46 (13%)	14 (10%)	5 (7%)
Neurologic	77 (22%)	50 (35%)	19 (27%)
Other	87 (25%)	24 (17%)	17 (24%)
Surgical	198 (19%)	66 (25%)	25 (22%)
Trauma	44 (8%)	43 (16%)	9 (8%)
Missing	32 (6%)	16 (6%)	10 (9%)
Admission source			
Direct admit	55 (11%)	28 (10%)	13 (11%)
Emergency room	133 (25%)	78 (29%)	27 (24%)
Floor	145 (28%)	55 (21%)	29 (25%)
ICU	14 (3%)	3 (1%)	1 (1%)
Operating room	98 (19%)	66 (25%)	25 (22%)
Transfer	47 (9%)	21 (8%)	9 (8%)
Missing	32 (6%)	16 (6%)	10 (9%)
APACHE III score: day 1	70 (30)	64 (26)*	69 (26)
Missing	25 (5%)	16 (6%)	10 (9%)
APS: day 1	57 (27)	53 (24)*	57 (24)
Missing	25 (5%)	16 (6%)	10 (9%)

Values are expressed as *n *(%), mean (standard deviation), or median (interquartile range). Statistical tests were performed between short-term ventilation and either DRG 541/542 or ventilation ≥ 21 days groups. *P *values by χ^2 ^test (for percentages), two-sided *t*-tests (for means), and Wilcoxon rank sum test (for medians). ^a^Comparisons are white versus non-white, home versus non-home, employed versus not employed, medical versus nonmedical diagnosis, and direct versus other admission. *P < 0.05 for comparison between short-term ventilation and DRG 541/542; ^†^*P *< 0.05 for comparison between short-term ventilation and ventilation ≥ 21 days. ADL, activity of daily living; APACHE, Acute Physiology and Chronic Health Evaluation; APS, Acute Physiology Score; DRG, diagnosis related group; IADL, instrumental activity of daily living; ICU, intensive care unit; MV, mechanical ventilation; SF-36, Short Form 36-item.

### Health outcomes

#### Mortality

**Figure 2:**
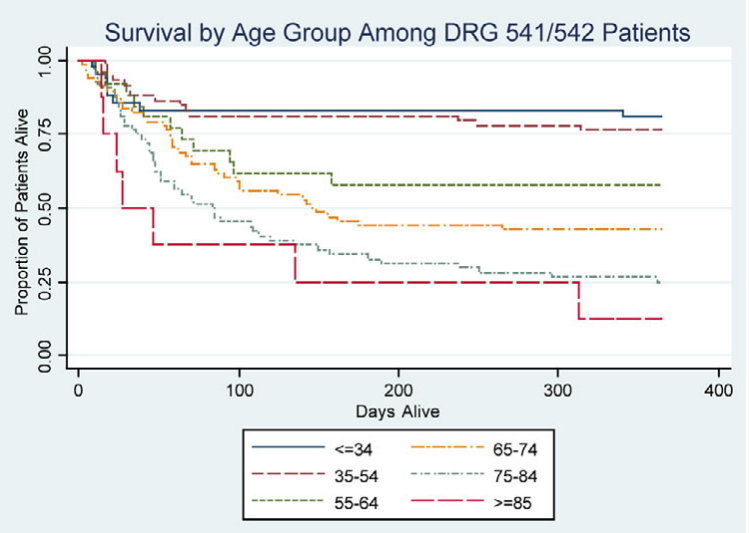
Survival by age group among DRG 541/542 patients. Kaplan-Meier plot demonstrating one-year survival stratified by age group among DRG 541/542 patients. Patients aged < 55 years have noticeably better overall survival than do older patients. Those < 55 years old also experience very low mortality rates after two months, whereas other age groups continue to die at relatively constant rates. *P *< 0.01 for comparisons between 65–74, 75–84, and ≥ 85 year age groups by logistic regression and adjusted for day one APS, preadmission IADLs, admission source, admitting diagnostic group, and preadmission Charlson score; *P *> 0.05 for comparisons between other age groups. APS, Acute Physiology Score; DRG, diagnosis related group; IADL, instrumental activity of daily living.

**Figure 3:**
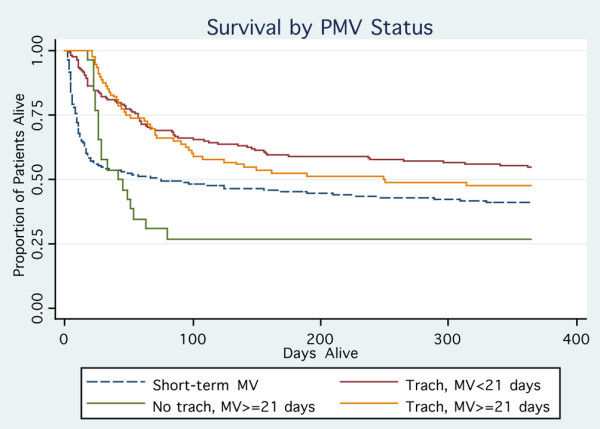
Survival among all patients by duration of ventilation and tracheostomy status. Kaplan-Meier plot demonstrating one-year survival by PMV status. The group with the best survival is those who were ventilated for < 21 days and who received a tracheostomy. Persons ventilated for at least 21 days but who did not receive a tracheostomy experienced the worst survival. Other groups had intermediate one-year survival. MV, mechanical ventilation; PMV, prolonged mechanical ventilation.

DRG 541/542 patients had significantly lower in-hospital mortality (20% versus 43%; *P *< 0.0001) and one-year mortality (48% versus 59%) compared with short-term ventilation patients (Table 2). Considering DRG 541/542 patients alone, mortality increased with patient age (Figure 2), although there were statistically significant adjusted one-year mortality differences only between patients in the 65–74, 75–84, and ≥ 85 year age groups (all *P *< 0.01). In-hospital and one-year mortality appeared higher for those ventilated for ≥ 21 days than for DRG 541/542 patients (statistical comparison not performed because of overlap between the groups). Mortality did not differ significantly between patient age strata (*P *= 0.30 by log-rank test) for patients ventilated ≥ 21 days. Patients ventilated for ≥ 21 days who did not receive a tracheostomy had particularly high mortality (Figure 3).

**Table 2:** Clinical outcomes and resource utilization by definition of prolonged mechanical ventilation

Short-term MV (*n *= 524)	DRG 541/542 (*n *= 267)	MV ≥ 21 days (*n *= 114)	
Mortality (cumulative)			
In-hospital	227 (43%)	53 (20%)*	36 (31%)^†^
(care limited)	114 (50%)	24 (45%)*	16 (44%)^†^
2 months	257 (49%)	74 (28%)*	40 (35%)
6 months	289 (55%)	115 (43%)*	61 (54%)
12 months	308 (59%)	127 (48%)*	65 (58%)
Discharge disposition			
Home	90 (17%)	19 (7%)*	5 (4%)^†^
Rehabilitation facility	111 (21%)	77 (29%)	27 (24%)
Nursing home	81 (15%)	60 (22%)	28 (25%)
Ventilator facility	0 (0%)	45 (17%)	9 (8%)
Other hospital	15 (4%)	13 (5%)	9 (8%)
Dead	227 (43%)	53 (20%)	36 (31%)
Status at 1 year			
Home	196 (37%)	134 (50%)*	47 (41%)
Rehabilitation facility	4 (1%)	2 (1%)	0 (0%)
Nursing home	16 (3%)	4 (2%)	2 (2%)
Dead	308 (59%)	127 (48%)	65 (57%)
Location of death			
Home	15 (5%)	3 (2%)	0 (0%)
Rehab facility	2 (1%)	1 (1%)	0 (0%)
Nursing facility	27 (9%)	24 (19%)	5 (8%)
Ventilator facility	1 (< 1%)	9 (7%)	2 (3%)
Hospital	262 (85%)	89 (70%)	57 (87%)
Other	0 (0%)	1 (1%)	1 (2%)
Ventilator days	6 (4, 9)	16 (10, 24)*	27 (23, 36)^†^
Reintubated	36 (7%)	46 (17%)*	25 (22%)^†^
Ventilator days before			
Tracheostomy	-	10 (5, 14)	14 (10, 20)
*n*	0 (0%)	267 (100%)	88 (77%)
ICU length of stay	8 (5 to 12)	22 (14 to 31)*	30 (24 to 41)^†^
Hospital length of stay	15 (9 to 21)	29 (22 to 38)*	39 (30 to 52)^†^
Hospital costs/patient	$40,968 ($25,773 to 65,959)	$111,194* ($80,164 to 156,312)	$152,709^† ^($115,565 to 221,959)
Costs/hospital survivor	$120,054	$164,956*	$266,105^†^
Costs/1-year survivor	$165,075	$266,105*	$423,596^†^

Values are expressed as *n *(%), mean (standard deviation), or median (interquartile range). Statistical tests were performed between short-term ventilation and either DRG 541/542 or ventilation ≥ 21 days groups. *P *values by χ^2 ^test (for percentages), two-sided *t*-tests (for means), and Wilcoxon rank sum test (medians). Costs are presented in 2005 US$. *P < 0.05 for comparison between short-term ventilation and DRG 541/542;^†^*P *< 0.05 for comparison between short-term ventilation and ventilation ≥ 21 days. DRG, diagnosis related group; ICU, intensive care unit; MV, mechanical ventilation.

**Figure 4:**
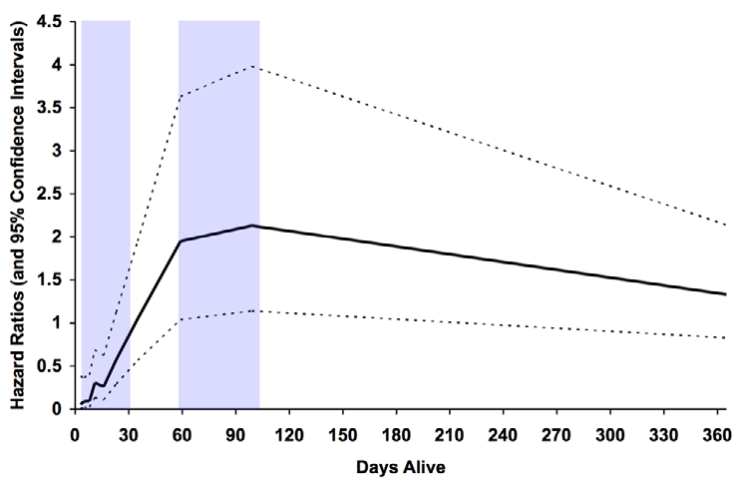
Hazard ratios for prolonged mechanical ventilation status over one year of follow up. Plot of hazard ratios (solid line) and 95% confidence intervals (dashed lines) for DRG 541/542 patients versus short-term mechanical ventilation patients, determined using a time-varying piecewise-constant nonproportional survival model. The shaded areas represent time periods with statistically significant hazard ratios. The hazard ratios vary over time, predicting an early (< 30 days after intubation) lower risk for death for DRG 541/542 relative to short-term ventilation patients, but a higher risk for mortality between days 60 and 100 as the slope of short-term ventilation mortality levels off (also see Figure 2). Hazard ratios are adjusted by day one APS, pre-admission Charlson score, age, and pre-admission ADLs. APS, Acute Physiology Score; ADL, activity of daily living; DRG, diagnosis related group.

The piecewise-constant time-varying survival model generated adjusted hazard ratios (95% confidence interval) for DRG 541/542 status compared with short-term ventilation over the course of follow up ranging from 0.05 (0.007–0.38) to 2.14 (1.15–3.99; Figure 4). Interestingly, hazard ratios for DRG 541/542 status ranged from 1.95 (1.05 to 3.63) to 2.14 (1.14 to 3.99) between 60 and 100 days after intubation, representing a higher risk for death, but they demonstrated no significant group-based differences thereafter.

#### Quality of life and functional status

**Figure 5:**
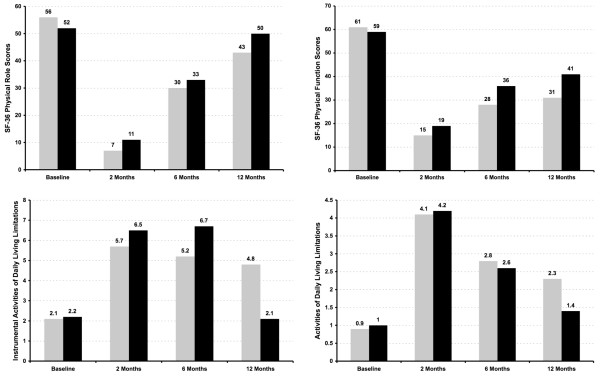
Quality of life and functional status over time for PMV patients. The gray bars represent PMV patients ventilated for ≥ 96 hours with a tracheostomy (DRG 541/542), and the black bars represent PMV patients ventilated for ≥ 21 days. Mean values are shown above the bars corresponding to scores on the SF-36 physical function and physical role scores as well as for limitations in both instrumental (IADLs) and basic (ADLs) activities of daily living. Because of the overlap of 88 persons in these two PMV groups, group-based statistical tests were not performed. ADL, activity of daily living; DRG, diagnosis related group; IADL, instrumental activity of daily living; PMV, prolonged mechanical ventilation; SF-36, Short Form 36-item questionnaire.

At one year, DRG 541/542 patients had significantly lower SF-36 physical function scores and more ADL and IADL limitations than short-term ventilation patients after adjusting for clinical characteristics (Table 3). Although DRG 541/542 patients had more profound early disability, they exhibited a similar, statistically significant rate of improvement in function recovery compared with those ventilated for shorter periods of time. Nonetheless, at one year the average DRG 541/542 patient had not returned to their preadmission functional status and was still receiving weekly care giving assistance. There were insufficient patient numbers to perform similar quality of life analyses between short-term ventilation patients and those ventilated ≥ 21 days. However, there were clinically important unadjusted functional status differences by PMV group (DRG 541/542 versus ventilation ≥ 21 days), although statistical testing was not done because of patient overlap (Figure 5).

**Table 3:** One-year health outcomes of hospital survivors by DRG 541/542 status

	Unadjusted^a^		Adjusted analyses for DRG 541/542 versus short-term MV^b^		
	
	Short-term MV	DRG 541/542	Between group difference (95% CI)	*t*	*P*
ADLs					
Preadmission	1.2 (1.9)	0.9 (1.8)	-0.3 (-0.6 to +0.04)	-1.70	0.09
2 months	2.5 (2.1)	4.1 (1.9)	1.6 (1.0 to 2.2)	5.81	< 0.0001
6 months	1.9 (2.1)	2.8 (2.1)	0.9 (0.3 to 1.5)	3.29	0.003
12 months	1.6 (2.0)	2.3 (2.1)	0.7 (0.2 to 1.2)	2.97	0.005
IADLs					
Preadmission	2.4 (2.8)	2.1 (2.7)	-0.4 (-0.9 to +0.1)	-1.41	0.16
2 months	4.8 (2.4)	5.7 (2.1)	0.9 (0.4 to 1.4)	3.60	0.0006
6 months	3.7 (2.6)	5.2 (2.4)	1.5 (0.8 to 2.2)	4.20	0.0003
12 months	3.4 (2.7)	4.8 (2.6)	1.4 (0.9 to 2.0)	4.86	< 0.0001
SF-36 physical function					
Preadmission	56 (38)	61 (37)	5 (-2 to +12)	1.39	0.17
2 months	29 (28)	15 (23)	-14 (-19 to -8)	-5.03	< 0.0001
6 months	42 (33)	28 (30)	-14 (-22 to -6)	-3.69	0.0006
12 months	46 (34)	31 (31)	-15 (-22 to -7)	-3.98	0.0002
SF-36 role physical					
Preadmission	53 (42)	59 (43)	6 (-3 to +14)	1.30	0.20
2 months	26 (30)	19 (25)	-7 (-13 to -1)	-2.48	0.01
6 months	44 (35)	36 (32)	-8 (-16 to -1)	-2.20	0.03
12 months	46 (36)	41 (34)	-5 (-14 to +4)	-1.21	0.23

Analyses for short-term mechanical ventilation (*n *= 312) and DRG 541/542 (*n *= 214) patients. ^a^Values from two-sample *t*-tests are expressed as means (standard deviation). ^b^Values are expressed as mean (95% confidence interval) based on linear-mixed effects models. Both unadjusted and linear mixed-effects models included imputed values and adjusted for day 1 APS, admitting service, pre-admission IADLs, pre-admission Charlson score, age ≥ 65 years, and education status. ADL, activity of daily living; DRG, diagnosis related group; IADL, instrumental activity of daily living; SF-36, Short Form 36-item questionnaire.

### Resource utilization

PMV patients defined by DRG 541/542 had significantly longer ICU and hospital length of stay, and their hospital costs were substantially higher than those ventilated for shorter periods of time (Table 2). Costs per one-year survivor were $165,075 for short-term ventilation patients, $266,105 for DRG 541/542 patients, and $423,596 for patients ventilated for ≥ 21 days. By identifying patients who received 'potentially ineffective care', or high-intensity (> $100,000 per hospitalization) medical treatment associated with early death (survival < 100 days), we were able to estimate short-term cost-effectiveness [33]. A total of 58 (22%) DRG 541/542 patients, 55% of whom were aged 65 years or older, and 47 (41%) of patients ventilated ≥ 21 days could be classified as having received potentially ineffective care. By comparison, fewer than 10% of the short-term ventilation patients received potentially ineffective care, even considering their 36% in-hospital mortality. Potentially ineffective care was associated with age, total days of ventilation, male sex, and number of preadmission IADLs (all *P *< 0.05 by logistic regression) but not with day one APS, admission source, or admitting service.

## Discussion

In this analysis of a large prospective cohort of mechanically ventilated patients, we found that patients who required PMV, particularly the elderly, remain at high risk for death during the first year after critical care and experience persistent, significant ICU-associated functional disability at great costs. This study also reveals that the two suggested definitions for PMV, DRG 541/542 and ventilation for ≥ 21 days, select cohorts with similar baseline clinical characteristics and trends in survival, disposition, and resource utilization. Importantly, however, PMV defined by ventilation for ≥ 21 days more specifically identifies patients who are outliers in resource consumption among ventilated patients. DRG 541/542 will remain a useful identifier for selecting PMV patients from large administrative databases, but the biases created by using this definition should be acknowledged in future studies.

Our analyses also provide compelling new observations about PMV patients related to their trajectories of post-discharge health outcomes and resource utilization. First, unlike patients ventilated for shorter periods of time, the majority of DRG 541/542 deaths occurred after hospital discharge and was disproportionately weighted toward the elderly. In addition to a high risk for postdischarge death, the average one-year DRG 541/542 survivor reported a notable burden of chronic illness reflected by two dependencies in basic functioning, five limitations in higher levels of functioning, and need for significant amounts of unpaid care giving assistance from family members. We also found that many PMV patients, particularly those ventilated for at least 21 days, received care with questionable short-term cost-effectiveness. These findings may help to clarify what PMV patients may experience regarding the general rate and magnitude of their functional recovery as well as reinforce others' concerns about the shifting of increasingly ill patients to posthospital care venues [4,14,18]. However, these observations also reflect the current difficulty in predicting PMV outcomes, because a physician's assessment that the patient has a reasonable chance of survival and basic functioning is inherent in their decision to place a tracheostomy.

Comparison of our findings with work by others is challenging because of differences in PMV definition and study design. Past research has shown one-year survival rates to range from 39% to 25%, similar to our patients [14,34]. Still others have described PMV hospital survival and reported contradictory findings regarding group-based mortality [9,35]. To our knowledge, however, one-year health outcomes of PMV patients have not been compared with concurrently enrolled non-PMV patients [36]. PMV patient costs in this study are similar to past work when adjusted to 2005 US$, although our assessments of potentially ineffective care are unique [4].

This study has limitations that are worth emphasizing. First, there was a significant amount of missing data due to death and inability to complete interviews, although we used novel statistical analyses to address these deficits. Because patients who could not complete interviews were more likely to have received PMV and also to have higher severity of illness scores, it is likely that this omission resulted in an underestimate of the PMV cohort's actual disability. Some may disagree with our choice to include both patient and proxy assessments of physical function in our analyses, although past experience with proxy-completed questionnaires has determined their reliability and validity [37]. Also, because of the unclear effect that refusals and eligibility factors during the enrollment of the original cohort had on our *post hoc *patient groups, our findings should be considered carefully. Finally, because this study was performed using a secondary source, it is susceptible to personal interpretational biases.

PMV provision and its associated $20 billion in annual inpatient costs have a profound effect on the health care system and those navigating within it [4]. Patients do not know what to expect from a course of PMV, and their family members have a high prevalence of depression and postdischarge care giving burden [18,38]. Also, clinicians struggle with PMV decision making because available prognostic models cannot match these patients' individuality [39]. Considering these observations, we believe that attention should be focused on developing PMV-specific health outcome prediction models, improving physician-family and physician-patient communication, and conducting formal economic analyses of PMV provision.

## Conclusion

PMV defined as ventilation for ≥ 21 days is more specific than DRG 541/542 (previously DRG 483) as marker of resource utilization and potentially ineffective care for true outliers of critical care, namely the chronically critically ill. However, the more sensitive term DRG 541/542 captures a group that nonetheless has persistent postdischarge deficits in functioning that are more profound than the disability of short-term ventilation recipients. Researchers should consider carefully the implications of these different PMV definitions based on the goals of future studies.

## Key messages

• Patients receiving mechanical ventilation for ≥ 21 days after acute illness have one-year mortality similar to that in patients receiving mechanical ventilation for shorter periods.

• Hospital costs for patients receiving PMV are substantially higher than for patients ventilated for shorter periods, and up to 41% of PMV patients receive potentially ineffective care.

• Identification of PMV patients using DRG 541/542, rather than the definition ≥ 21 days of mechanical ventilation, selects patients who have lower illness severity, lower mortality, and lower hospital costs.

• Despite having better baseline functional status than patients ventilated for shorter periods, DRG 541/542 patients have lower functional capabilities after one year.

## Abbreviations

ADL = activity of daily living; DRG = diagnosis related group; IADL = instrumental activity of daily living; ICU = intensive care unit; PMV = prolonged mechanical ventilation; SF-36 = Short Form 36-item questionnaire.

## Competing interests

The authors declare that they have no competing interests.

## Authors' contributions

CC conceived this secondary study, performed statistical analyses and interpreted data, and drafted the manuscript. SC interpreted data and drafted the manuscript. MO and JHL performed statistical analyses and drafted the manuscript. JG interpreted the data and drafted the manuscript. LC obtained funding for the original study, designed the original study, gathered data for the original study, supervised this study, and revised the manuscript critically. CC, SC, MO, JHL, and LC have given final approval of the version to be published.
